# Standard operating procedure for computing pangenome trees

**DOI:** 10.4056/sigs.38923

**Published:** 2010-01-28

**Authors:** Lars Snipen, David W. Ussery

**Affiliations:** 1 Biostatistics, Department of Chemistry, Biotechnology and Food Science, Norwegian University of Life Sciences, Ås, Norway; 2 Center for Biological Sequence Analysis, Technical University of Denmark, Lyngby, Denmark

## Abstract

We present the pan-genome tree as a tool for visualizing similarities and differences between closely related microbial genomes within a species or genus. Distance between genomes is computed as a weighted relative Manhattan distance based on gene family presence/absence. The weights can be chosen with emphasis on groups of gene families conserved to various degrees inside the pan-genome. The software is available for free as an R-package.

## Introduction

Currently, there are about a thousand sequenced prokaryotic genomes in GenBank, and several thousand more are in various stages of completion. For many bacterial species, sequenced genomes from several different strains are available, opening the possibility to study pan-genomes or supra-genomes. The pan-genome of a species or genus, as opposed to the genome of a single strain, is defined as the union of all gene families found at least once in a genome within that species or genus [1,2]. Studying the diversity within pan-genomes is of interest for the characterization of the species or genus. Low pan-genome diversity could be reflective of a stable environment, while bacterial species with substantial abilities to adapt to various environments would be expected to have high pan-genome diversity. Visualizing the relations between genomes within pan-genomes could also be helpful in establishing a picture of the degree of horizontal gene transfer (HGT), as well as aid in the understanding of phenotypic differences.

Diversity between genomes is often displayed in the form of trees. Over the past decade several procedures have been proposed for constructing trees from more or less whole-genome data [3,4]. Many strategies have been employed, and two major approaches are sequence-based and gene-content based trees. Sequence based trees include super-trees and phylogenomic trees, and their construction is based more or less directly on sequence alignments and evolutionary distances known from classical phylogenetics [5-7]. The gene content trees use as data the presence/absence of genes in the various genomes, and compute distance between genomes from such data [8,9]. The pan-genome tree described here would naturally be categorized amongst the gene-content trees.

It should be noted that the vast majority of genome-trees are constructed with the ultimate goal of reconstructing evolution. As for the gene-content trees, this has the effect that a separation between orthologs and paralogs is crucial, and HGT is considered to be noise that ideally should have no impact on calculation of distances between genomes (in the case of distance based trees). There are, however, other reasons for building trees. In applied sciences like medicine or agricultural sciences, a functional relation is as important as evolutionary distance. Admittedly, a good reconstruction of evolution can be very helpful to unravel the functional relations, but discarding HGT as noise in order to present a clean view of history is clearly a mistake in this context. The pan-genome tree we describe here is intended to display, in a hierarchical tree-like structure, the functional relationship between a “snapshot” set of sequenced genomes.

## Requirements

The software is implemented in R, which is a freely available computing environment, see http://www.r-project.org. A package for microbial pan-genomics is under construction, and a pre-release version is available upon request from the corresponding author. The computation of gene families mentioned in this paper is based on BLAST, which is available at ftp://ftp.ncbi.nih.gov/blast/.

## Procedure

### Gene families

Sequences are grouped into gene families based on sequence similarity. A FASTA formatted file with all protein sequences for one genome is BLASTed against similar sequences for all genomes, including itself. Two sequences are in the same gene family if there are significant alignments between them when either sequence is used as query, and when both these alignments span at least 50% of the length of the query sequence and contain at least 50% identity ([1]).

The gene family results are represented in a pan-matrix ***M***, where each row corresponds to a genome and each column to a gene family. Element *M_i,j_* is 1 if gene family *j* is present in genome *i,* or 0 if not. Hence, each row of ***M*** is a sequence of binary digits which we refer to as the pan-genome profile of the corresponding genome. When we use the term “genes” below we actually mean gene families.

### Pan-genome trees

The genome trees are formed on the basis of distance between pan-genome profiles. We use a relative Manhattan distance, *i.e.* the distance between genome *i* and *k* is


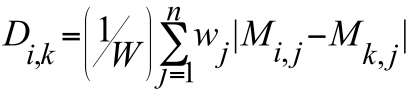


Where *n* is the total number of gene families, *w_j_* is some gene family specific weight and *W* is the sum of these weights. As default *w_j_=1* for all *j*, but some genes may be down-weighted, as described below. This distance describes the proportion of the pan-genome in which genome *i* and *k* differ. A frequently used distance for phylogenetic gene-content trees is the Jaccard distance [10]. Considering genomes A and B, genes are either i) present in both, ii) present in A and absent in B, iii) absent in A and present in B or iv) absent in both. The Jaccard distance is 1 minus the number of genes in class i) divided by the sum of genes in class ii) and iii). The Manhattan distance we use above is the sum of genes in ii) and iii) divided by the sum of all genes. A similar, unweighted, distance was also used in [11] in their construction of the pan-genome tree.

Using this distance measure, trees can be formed by hierarchical clustering. We have employed an average linkage, corresponding to the Unweighted Pair-Group Method with Arithmetic mean (UPGMA) algorithm; UPGMA has been previously used in the building of phylogenetic trees.

Bootstrapping is frequently used to illustrate the stability of the branching in a tree. We have implemented this by re-sampling gene families, *i.e.* columns of the pan-matrix, and re-clustering these data. The bootstrap-value for a split is the percentage of the re-sampled trees having a similar node, i.e. with the same two sets of leaves in the branches.

### Gene family weights

The core genes, *i.e.* the gene families present in all genomes, contribute to no difference between genomes, and could be discarded, *i.e.* given weight zero. Other gene families may also be down-weighted. Genes found in only one single genome, referred to as ORFans, are often dubious and can be the product of over-sensitive gene finders. Hence, giving such genes zero weight could improve the robustness of the tree to these types of errors.

**Figure 1:**
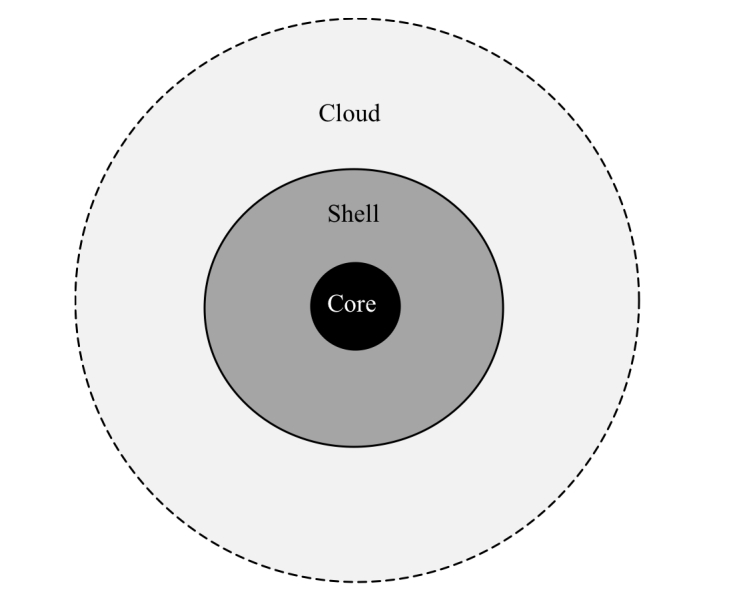
The bacterial pan-genome can be divided into the core (genes always occurring in any genome inside the pan-genome) the shell (genes frequently occurring) and cloud (rarely occurring genes).

It has been observed that genes could be divided into classes depending on their degree of conservation within the pan-genome. This is the basis for the use of mixture models to predict pan-genome size [12,13]. In [14] the bacterial pan-genome was divided into three major categories, core, shell and cloud, as illustrated in Figure 1.

**Figure 2:**
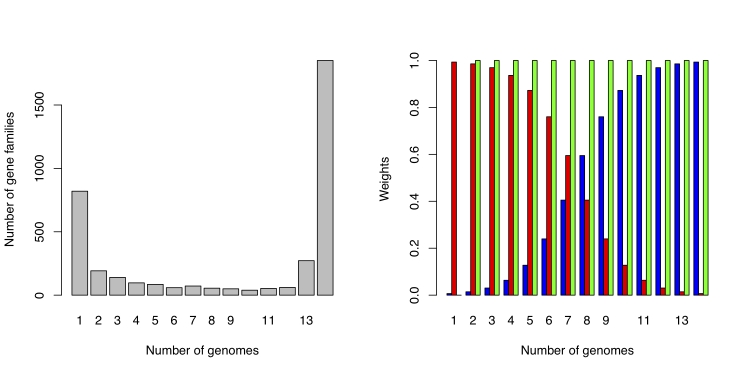
The left panel shows as an example the number of gene families found in 1, 2,…,14 genomes of the 14 completed genomes of *Staphylococcus aureus* downloaded from NCBI in July 2009. The right panel illustrates three possible weighting schemes. The green bars give weight 1.0 to all gene families except the ORFans, *i.e.* those gene families only present in one genome, who get weight 0.0 (discarded). The blue bars give a gradually higher weight to the gene families found in the majority of the genomes, the shell. The red bars illustrate the opposite strategy, emphasizing the cloud. All gene families found in the same number of genomes get the same weights.

The Shell represents the genes found in the majority of the genomes, and the corresponding Cloud consists of genes only observed in a minority of the genomes. Weights can be designed to emphasize both these types of gene families. Figure 2 illustrates these weighting strategies.  The size of the cloud and shell can be significantly larger than the core genome [13], reflecting the diversity (or lack thereof) of various types of bacteria in different ecological niches.  For example, the shell and cloud would be expected to be larger for *Actinobacteria* and other organisms that produce secondary metabolites.  Further, the pan-genomes of phyla could contain specific pathways which are phylum- or class-specific (*e.g.* polyketides type I and II pathways, aminoglycosides, non-ribosomal peptides, β-lactams, *etc*), that would be part of phylum specific shells.  On the other hand, pathogenic, parasitic and commensal species that are not routinely found in the environment could have smaller clouds.

## Implementation

Standard settings for BLASTp were used, except the E-value cutoff, where we use 10^-5^. A more liberal cutoff will have very small effects on the final results, but will slow down the procedure significantly by producing a lot of poorer alignments in addition to the best alignments. Since the BLASTing and parsing of BLAST results is the computational bottleneck of this procedure we have found this cutoff appropriate. The remaining computations and plotting have been implemented in R as part of a package for microbial pan-genomics.

### Pan-genome tree versus 16S phylogenetic tree

**Figure 3:**
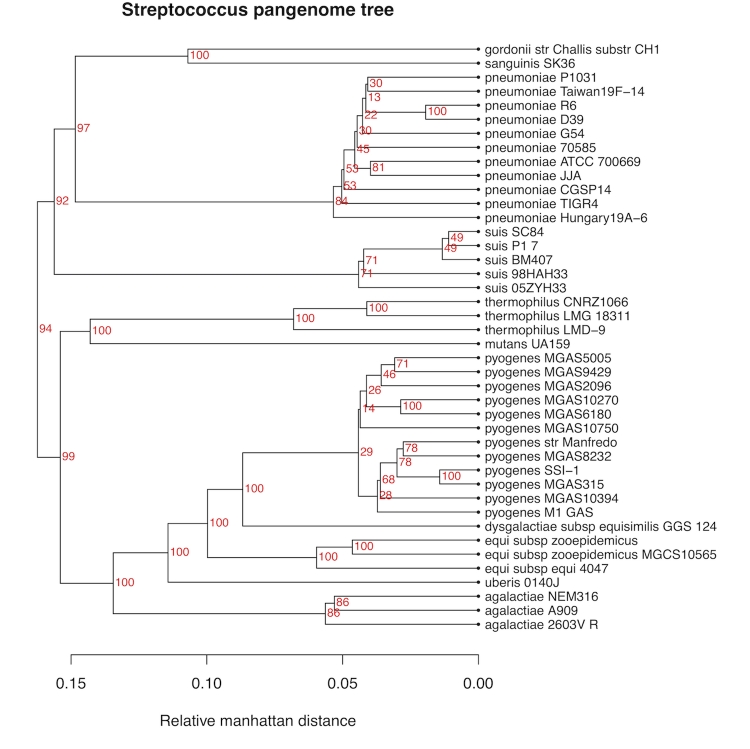
Pan-genome tree for the genus *Streptococcus*. The red numbers are bootstrap values (percentages).

Figure 3 show a pan-genome family tree for the genus *Streptococcus*, based on 42 completed genomes downloaded from NCBI in August 2009. We have used this genus as an example, because it contains several species with multiple completed genomes. All genomes within each species cluster together, without exception, and further the resolution is good enough to distinguish smaller differences among strains within the species.

**Figure 4:**
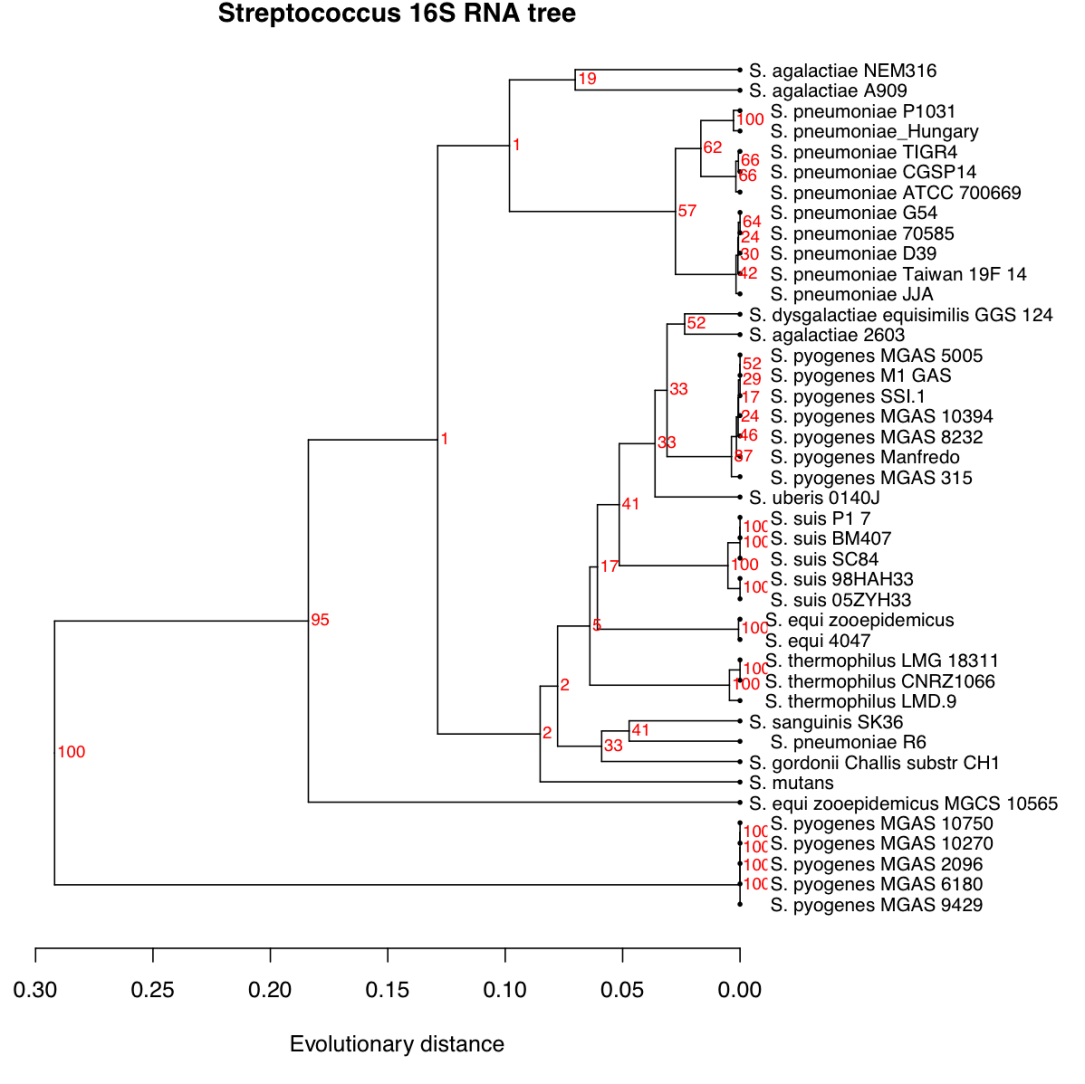
A tree for the same genomes as in Figure 3, but computed from distances based on multiple alignment of the 16S ribosomal RNA sequence from each species. The tree is constructed by UPGMA to make it comparable to that in Figure 3.

In Figure 4 we have, for comparison to Figure 3, included another tree for the same genus, based on the more traditional approach of computing evolutionary distances from the multiple alignment of the 16S ribosomal RNA sequences of each genome. Here we typically see extremely small distances between many strains, combined with some bigger distances, giving a lower resolution. Also, *S. pyogenes* is divided into two very different clusters with 7 and 5 genomes in each. The smaller cluster of *S. pyogenes* strains all share an almost identical annotation of the 16S sequences differing in length from all other streptococci. In the pan-genome tree of Figure 3 this division of *S. pyogenes* strains is not supported. Also the *S. agalactiae* genomes are no longer clustered in the 16S tree, and the strain *S. pneumoniae* R6 is also found separated from all other *S. pneumoniae* strains. The 16S tree was constructed using UPGMA in order to make it comparable to that in Figure 3. UPGMA is in general not accepted as a proper way of re-constructing phylogenetic trees, but a tree built by neighbor-joining verified the separation of strains, even if distances between nodes changed (not shown).

### Effect of weights

**Figure 5:**
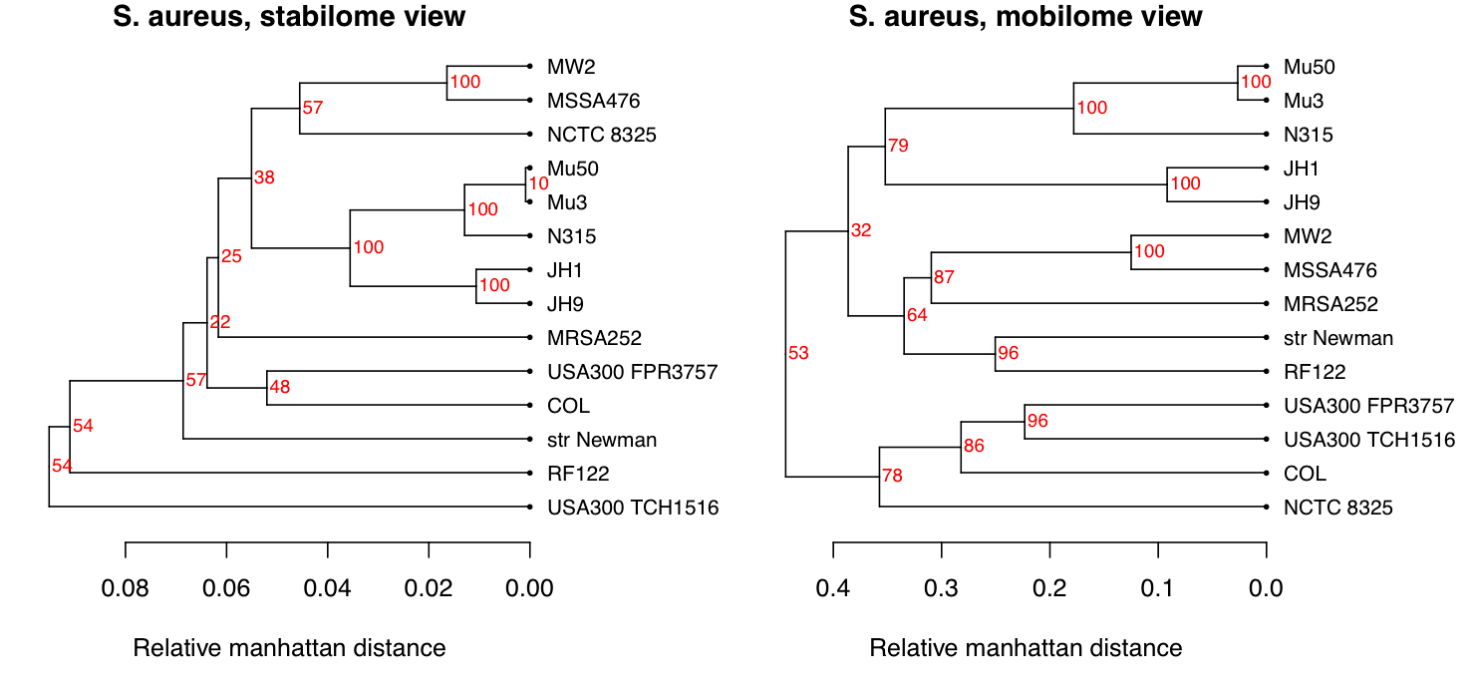
The left panel show the pan-genome family tree for the 14 strains of *Staphylococcus aureus* completed as NCBI. Here the weights have been chosen according to the blue bars in Figure 1, i.e. the “stabilome” genes have been emphasized. In the right panel, the same data have been used, but weights are now chosen to emphasize the “mobilome” genes. In both cases ORFans have been discarded.

In Figure 5 we illustrate different choices of weights. Here we have used data for a single species, *Staphylococcus aureus*. Annotated proteins for all completed genomes of this species were downloaded from NCBI. Note that there are some differences in clustering - for example, the two USA300 strains, which are community-acquired methicillin-resistant strains [15] that would be expected to be similar, are not as close in the shell, but cluster together when more weight is given to the “cloudy part” of the pan-genome. Thus, these two strains are not very similar when we consider the *S. aureus* typical part of the genomes, but become more alike when we instead focus on the rarely occurring, more strain-specific (accessory) genes.

## Discussion

We present here the pan-genome tree as a standard operating procedure in the pan-genomic toolbox. It is a whole-genome tree not unlike many other gene-content trees, but with the emphasis on describing functional differences between closely related genomes, within a species or genus. Examples of successful use of variants of such trees are [11] and [16].

The distance between genomes is the relative Manhattan distance between pan-genome profiles. Two genomes are similar not only by sharing the same genes as defined by the Jaccard distance, but also by lacking the same genes. The latter is meaningful inside a pan-genome where all the genes could in principle be present. When looking for differences in phenotype those parts of the “machinery” which are absent are just as important as those that are present. In [17] an estimate of shared absence was introduced by including a third reference genome when comparing two genomes of interest. In our case the pan-genome plays the role as the reference genome.

The weights illustrated in Figure 2 are only a selection out of a long range of possible choices. Discarding ORFans, and emphasizing the shell or cloud, are, however, strategies with a meaning. Weighted distances in gene-content trees have been used before*, e.g.* [18]. Two of their weighting strategies, termed prevalence-weighted and rarity-weighted trees, are in principle similar to what we call shell and cloud strategies.

A pan-genome profile of a genome is a vector of 1s (present) and 0s (absent) with N elements if the pan-genome has N gene families. In [10] the term conservation profile was used for a similar vector, but with one vector for each gene sequence in each genome. Merging these sequence-specific conservation profiles into one pan-genome profile for the entire genome is in principle what is done when gene families are computed and the pan-matrix constructed. We compute gene families in a simple way, using BLAST and a simple cutoff-rule. This will have to change in near future, because the alignment of all-against-all is not a computationally feasible solution as the number of genomes grows. Computing gene families by BLASTing against a database like COG [19] has been a common strategy and Wolf *et al*. [8] concluded that gene-content trees based on presence/absence of such gene families resulted in a grouping of genomes based on phenotype. However, groups of orthologs, like the COGs, are often large and diverse and in our experience give too few and too large gene families to achieve good resolution when clustering closely related genomes. We are currently working on improvements of this.
